# Targeted gluteal exercise versus sham exercise on self-reported physical function for people with hip osteoarthritis (the GHOst trial – Gluteal exercise for Hip Osteoarthritis): a protocol for a randomised clinical trial

**DOI:** 10.1186/s13063-018-2873-3

**Published:** 2018-09-20

**Authors:** Adam Ivan Semciw, Tania Pizzari, Stephanie Woodley, Anita Zacharias, Michael Kingsley, Rod A. Green

**Affiliations:** 1 Department of Rehabilitation, Nutrition and Sport; La Trobe University, Bundoora, VIC Australia; 2 School of Health and Rehabilitation Sciences, The University of Queensland, St Lucia, QLD Australia; 3 Department of Anatomy, School of Biomedical Sciences, University of Otago, Dunedin, New Zealand; 4 Department of Pharmacy and Applied Science, La Trobe University, Bendigo, VIC Australia; 5 Exercise Physiology, La Trobe Rural Health School, La Trobe University, Bendigo, VIC Australia

**Keywords:** Hip osteoarthritis, Exercise, Buttocks, Gluteal, Gait, Clinical trial

## Abstract

**Background:**

Clinical practice guidelines recommend exercise as the first line of management for hip osteoarthritis, yet high-quality evidence from Cochrane reviews suggest only slight benefits for pain and physical function; and no benefit on quality of life (low-quality evidence). However, the scope of physical impairments identified in people with hip osteoarthritis may not have been adequately addressed with targeted rehabilitation options in previous randomised controlled trials (RCTs). Potential targeted options include gait retraining to address spatio-temporal impairments in walking; motor control training to address deep gluteal (gluteus minimus) dysfunction; and progressive, high-intensity resistance exercises to address atrophy of the gluteal muscles. The aim of this study is to investigate the effect of a targeted gluteal rehabilitation programme that incorporates gait retraining, motor control and progressive, high-intensity resistance-strength training, to address physical activity levels and self-reported physical function in people with mild to moderate disability from hip osteoarthritis.

**Methods:**

Ninety people diagnosed with mild to moderately disabling hip osteoarthritis will be recruited and randomised to receive one of two exercise programmes (sham or GHOst programme). Interventions will be 12 weeks in duration, with weekly, supervised physiotherapy sessions, and daily home exercises. Both groups will receive standardised education. Outcomes will be assessed at baseline, 7 weeks, 13 weeks (primary time-point) and 25 weeks. The primary outcome will be self-reported physical function measured with the Western Ontario and McMaster Universities Osteoarthritis Index (WOMAC). Secondary outcomes include physical activity measured with a tri-axial accelerometer, physical function tests, self-reported physical activity, isometric hip-muscle strength tests, hip-related patient-reported outcome measures, pain thoughts and depressive symptoms, quality of life, global rating of change, gluteal-muscle activity (electromyography (EMG)) and gluteal-muscle size and adiposity (magnetic resonance imaging (MRI)).

**Discussion:**

This will be the first study to compare a targeted gluteal rehabilitation programme to a sham exercise programme. The targeted GHOst programme includes exercises designed to address gait impairments as well as gluteal-muscle atrophy and dysfunction.

**Trial registration:**

Australian New Zealand Clinical Trials Registry, ID: ACTRN12617000970347. Registered retrospectively on 5 July 2017. Protocol version 3.0.

**Electronic supplementary material:**

The online version of this article (10.1186/s13063-018-2873-3) contains supplementary material, which is available to authorized users.

## Background

Osteoarthritis is a chronic, debilitating condition affecting many people worldwide. In 2016, approximately 4% of the global population had a diagnosis of osteoarthritis, with the prevalence reaching 9% in countries such as the United States, Germany and Canada [1]. Osteoarthritis typically presents in the weight-bearing joints of the lower limb such the hip and knee [2]. Symptoms in people with hip osteoarthritis usually deteriorate slowly [3], and this likely contributes to a reduction in physical function [4, 5] when undertaking simple activities such as walking. The symptoms and reduction in physical activity are thought to contribute to elevated Body Mass Index (BMI), weakness, stiffness, psychological distress, poorer quality of life [6], and greater personal and societal burden [7, 8].

Clinical practice guidelines recommend exercise and education as first-line management for osteoarthritis of the hip or knee [9–13]. The ultimate goal of exercise participation in people with osteoarthritis is to improve symptoms, physical function and quality of life. Theoretically, the improvements obtained with exercise are achieved by addressing bio-physiological (e.g. muscle strength, joint stability, cartilage integrity) and psychosocial impairments present in this patient group [4, 14–18]. A Cochrane review and meta-analysis concluded that there is moderate- to high-quality evidence that exercise has moderate benefits for pain and physical function, and slight benefits for quality of life in people with *knee* osteoarthritis [19]. However, exercise interventions for those with *hip* osteoarthritis appear to be less efficacious. High-quality evidence from a Cochrane review and meta-analysis suggests that exercise has only slight benefits for pain and physical function in people with hip osteoarthritis [20]; and no improvement in quality of life (low-quality evidence) [20]. A more recent, high-quality randomised controlled trial (RCT) supplements these findings to suggest that exercise combined with manual therapy is no better than a sham intervention (turned-off ultrasound) for improving pain and physical function in this patient group [21]. It is possible that the principles of exercise training programmes (frequency, intensity, type, duration and progression) have not been optimised to target the specific impairments associated with hip osteoarthritis, and these training principles are an important consideration for clinical prescription and future research [20, 22, 23].

Targeting specific impairments might be important to optimise outcomes of rehabilitation for people with hip osteoarthritis. Peak hip-abductor isometric strength, and hip-adduction moment during gait (a surrogate measure of hip-abductor strength) are significantly associated with pain while walking in people with hip osteoarthritis [24]. Furthermore, hip-abductor muscle atrophy is present in those with hip osteoarthritis, and this is associated with radiological and clinical severity [25, 26]. The use of progressive, high-intensity resistance-strength training for the hip abductors may potentially have a significant impact on symptoms during functional tasks like walking, and ultimately affect physical function and quality of life. There are, however, no studies that have investigated high-intensity resistance-strength training in this population [14, 20, 21]. In addition, individuals with symptomatic hip pathology present with biomechanical [27] and spatio-temporal [28] gait impairments that could potentially be addressed with rehabilitation. For example, reduced peak hip extension during gait is evident in early hip pathology [29] as well as established hip osteoarthritis [27, 30]. It has been theorised [31, 32] that loss of terminal hip extension reduces the stimulus of the deep anterior hip muscles (e.g. gluteus minimus (GMin)) to contract [33]. Weak or inefficient anterior hip muscles may consequently leave the anterior-superior hip joint vulnerable to further shearing, injury and degeneration [34, 35]. Indeed, GMin atrophy is present during end-stage hip osteoarthritis [26, 36], with a particular susceptibility of anterior GMin [37], and persists following hip surgery [38, 39]. It is, therefore, important to consider gait retraining and motor control strategies to correct for these biomechanical and muscle impairments.

Gait retraining has been recommended in recent clinical guidelines for the management of hip osteoarthritis [11]; however, this is based on weak evidence including expert opinion. There are currently no studies that include gait retraining during rehabilitation in people with hip osteoarthritis. Clearly, the scope of rehabilitation options for targeting specific impairments in people with hip osteoarthritis has not been fully explored. Potential options include, high-intensity resistance-strength training, gait retraining and motor control strategies targeting muscle segments (e.g. GMin anterior) that are known to atrophy in this population [37].

Using a parallel RCT design, the aim of this study is to compare a targeted gluteal rehabilitation programme that incorporates gait retraining, motor control and progressive, high-intensity resistance-strength training, to a sham exercise intervention for addressing self-reported physical function in people with mild to moderately disabling hip osteoarthritis.

## Methods

This study will be reported according to the Consolidated Standards of Reporting Trials (CONSORT) 2010 guidelines [40] and the protocol developed according to Standard Protocol Items: Recommendation for Interventional Trials (SPIRIT) (Additional file 1) [41, 42]. The study design and protocol follows the OARSI clinical trials recommendations for the conduct of clinical trials for hip osteoarthritis as set out in Lane et al. [43]. This study will compare the effect of a 12-week, targeted, physiotherapy-supervised gluteal exercise programme, with a 12-week physiotherapy-supervised, sham exercise programme in people with hip osteoarthritis. The trial has been registered on the Australian New Zealand Clinical Trials Registry (ACTRN12617000970347) with a Universal Trial Number (UTN) U1111–1192-1408. Where appropriate, approval for trial modifications will be sought by the relevant ethics committee and updated on the ANZCTR. The study will take place in three Australian cities (Bendigo, Melbourne, Brisbane) and one New Zealand city (Dunedin) involving physiotherapy clinics across the public and private sectors.

### Ethical approval and consent

This trial adheres to the principles of the Declaration of Helsinki, and has received ethical approval from the Bendigo Health Care Group Human Research Ethics Committee (HREC/17/BHCG/3); La Trobe University HREC; The University of Queensland (20,170,001,541/HREC/17/BHCG/3) and Otago University HDEC (17/STH/205). All participants will provide informed, written consent before commencing the study.

### Design

**Fig. 1:**
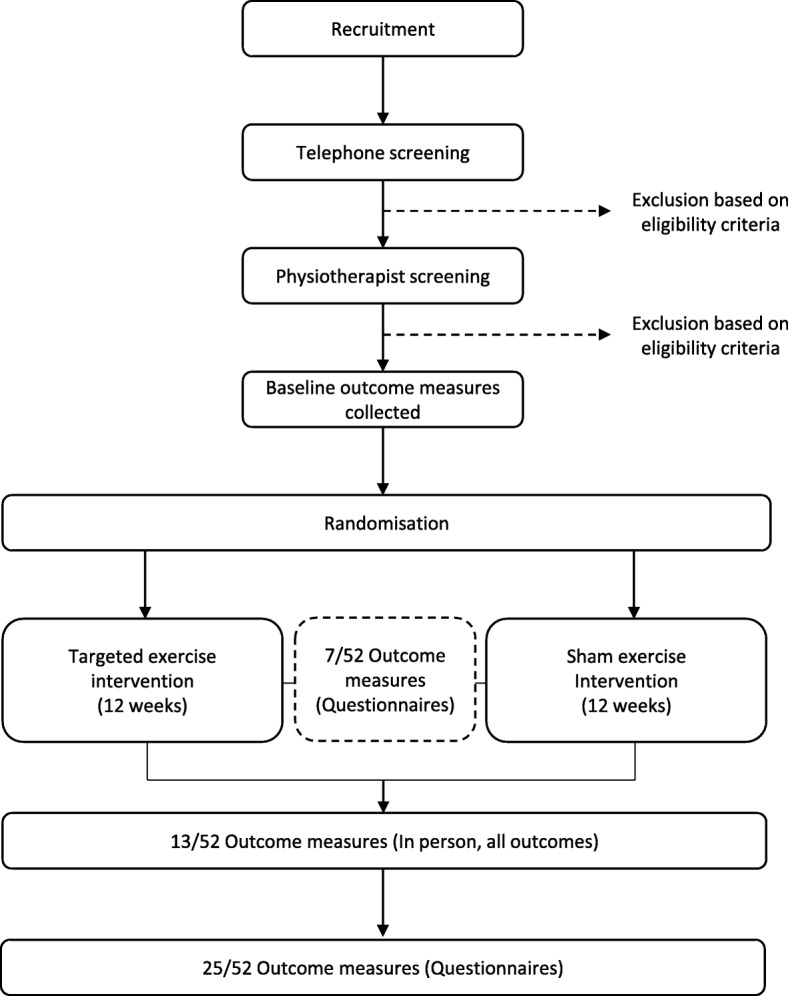
Flow of participants through the study

This is a two-arm, assessor- and participant-blind, parallel, randomised clinical trial, with a 12-week intervention period and outcomes measured at baseline, 7 weeks, 13 weeks and 25 weeks (Fig. 1).

### Participants

#### Recruitment

Potential participants with hip osteoarthritis will be recruited via local print media and through advertising flyers distributed through participating health providers and community notice boards. Further advertising through social media and radio and online advertising service will be conducted. Interested volunteers will contact chief investigators (RG, AS, TP, SW) at one of the four participating sites via telephone or email and will be screened (by telephone) for eligibility.

#### Eligibility

The inclusion criteria are as follows: mild to moderate disability from idiopathic (primary) hip osteoarthritis in accordance with the American College of Rheumatology [44] as defined by:

(i) Aged > 35 years
(ii) Pain in the hip or groin for more than 3 months
(iii) Pain intensity over the past week of ≥ 30 or higher on a 100-mm visual analogue scale (VAS) during functional tasks like walking, climbing stairs or climbing in/out of a car
(iv) Radiographic confirmation of hip osteoarthritis with a Kellgren-Lawrence score ≥ 2 [45]
(v) Mild to moderate disability indicated by:
  - Oxford Hip Score of 25–45 indicating mild to moderate disability [46, 47]
  - Still able to reciprocally ascend and descend 10 stairs unaided [48]
  - Still able to safely walk one city block, and
  - Able to jog 5 m
(vi) Satisfactory completion of an adult pre-exercise screening tool (https://www.essa.org.au/wp-content/uploads/2011/09/Screen-tool-version-v1.1.pdf)

Exclusion:

(i) Other musculoskeletal lower limb or back conditions requiring assessment or treatment in the last 6 months
(ii) Primary complaint of gluteal tendinopathy (clinical diagnosis), low back pain or referred back pain (Additional files 2 and 3)
(iii) History of hip trauma or surgery on either the affected or unaffected side
(iv) Known knee joint pathology that may impact on the ability to perform the intervention or reduced knee range of motion (< 90° flexion)
(v) Corticosteroid use (oral or intra-articular) in the past 3 months
(vi) Neurological impairment or condition affecting lower-limb function
(vii) Conditions or factors affecting ability to take part in the exercise intervention, e.g. unavailable for a 12-week intervention period, routine use of gait aids, uncontrolled hypertension, or morbid obesity (Body Mass Index > 40)
(viii) Systemic inflammatory disease (e.g. rheumatoid arthritis)
(ix) Unable to write, read or comprehend English

In addition, participants at one site (Bendigo; total of 30 participants) will be asked to complete a magnetic resonance imaging (MRI) scan and undergo fine-wire electromyography (EMG) of the GMin and gluteus medius (GMed) muscles. These volunteers will be ineligible to participate if they have any contraindication to fine-wire EMG (e.g. fear of needles, taking blood-thinning medication) or MRI (e.g. pacemaker, claustrophobia).

### Study procedure

#### Telephone screening

Interested volunteers will initially be screened over the telephone by one of the chief investigators at each of the four study sites. Telephone screening will aim to assess and exclude potential participants who do not fulfil the eligibility criteria. Reasons for exclusion at this stage include, but are not limited to; a clear diagnosis other than hip osteoarthritis; inability to complete a 12-week intervention programme at a trial physiotherapy clinic; Oxford Hip Score outside the range of 25–45; unable to walk a city block, ascend/ descend a set of 10 stairs, or jog 5 m; pain < 30 mm on a VAS during functional tasks; routine use of gait aids.

#### Physiotherapy assessment

Potential participants who satisfy the initial telephone screening will be invited to attend a physiotherapy screening examination. This screening session will include a physical examination to exclude gluteal tendinopathy and lumbar spine pain as the primary source of hip pain; and to exclude knee pathology that may affect their ability to complete the exercise programme. Screening for gluteal tendinopathy (clinical diagnosis) will be completed using the following protocol (Additional file 2): (1) tenderness to palpation over the greater trochanter (high sensitivity [85.7 (66.4–95.3)] suggesting that a negative test would rule out the condition [49]), in combination with (2) pain reproduction with the flexion, abduction, external rotation test (FABER) [49, 50] and (3) one other clinical test associated with gluteal tendinopathy (resisted external de-rotation test, resisted abduction test, single-leg stance test (30 s) [49, 51]. Lumbar spine referral as a primary source of pain will be screened by the following criteria (Additional file 3): (1) reproduction of hip symptoms with repeated lumbar flexion, lumber extension, or quadrant testing and (2) reproduction of hip symptoms with passive straight leg raise to 45° hip flexion. The physiotherapist will also assess passive knee range of motion to ensure > 90° of pain-free movement in order to screen for serious knee pathology. If potential participants satisfy the remaining eligibility criteria, they will once again be instructed on the nature of the study, offered the opportunity to ask questions about the study, and invited to provide written, informed consent (Additional file 4).

### Randomisation, allocation and blinding

A randomisation schedule will be generated by an investigator (MK) who will have no contact with participants for the duration of the trial, including participant screening, baseline assessment, intervention, or other outcomes assessment. A web-based randomisation programme (https://www.randomizer.org/) will be used to generate the randomisation schedule, with a 1:1 allocation ratio, stratified by site. Group allocations will be concealed in serially numbered, opaque, sealed envelopes for each site. These envelopes will be posted to the trial physiotherapist at each site who will not be involved in participant screening or recruitment. The trial physiotherapist will open the envelopes sequentially according to participant number to determine the participant’s group allocation prior to their first appointment (after eligibility screening, enrolment and baseline testing has been completed).

#### Assessor blinding

All outcome measures will be assessed by a research assistant at each site who will be blind to participant group allocation. Participants will be instructed not to divulge any aspect of their intervention to the research assistant conducting follow-up assessments.

#### Participant blinding

Participants will be blinded to group allocation (sham or Gluteal exercise for Hip Osteoarthritis (GHOst) protocol). They will be advised that they have an equal chance of being allocated to the either protocol. Participants will also be blind to the study hypothesis, so they are unaware which of the interventions is ‘active’.

#### Physiotherapy blinding

It is not possible to blind the trial physiotherapists to group allocation. Trial physiotherapists will, however, not be involved in assessment outcome measures.

### Interventions

Participants will be randomly allocated to the GHOst intervention or the sham intervention. Both groups will receive standardised education for hip osteoarthritis delivered by a trial physiotherapist and supplemented with handouts. Education will focus on an understanding of the condition and general advice on physical activity. Participants will be asked to refrain from other active, supervised (e.g. from a health professional) non-pharmacological rehabilitation for their hip osteoarthritis for the duration of the trial.

Both groups (GHOst and sham) will receive once weekly, supervised physiotherapy sessions and a self-managed exercise programme over 12 weeks. The self-managed home exercise programme will consist of the same exercises prescribed during the supervised sessions. The initial physiotherapy session will be a one-to-one session for 1 h, incorporating education and exercises, delivered by the trial physiotherapist. The additional supervised sessions will be 30 min and conducted in an individual or group setting (i.e. one to three participants per session), based on availability of participants and scheduling of the trial physiotherapist. Participants will only attend group sessions with other participants who have been randomised to the same intervention. Whether participants attend group or individual sessions will be documented by the trial physiotherapist, and accounted for as a potential covariate in statistical analysis. Daily exercise adherence (frequency and duration) will be monitored with an exercise diary [52].

#### GHOst protocol: targeted gluteal intervention

The GHOst protocol is composed of three phases. Gait retraining, motor control (anterior hip stability) and pelvic stability with global, high-intensity resistance-strengthening. Exercises within each phase are progressed based on quality (judged by the trial physiotherapist), symptoms (pain not exceeding 5/10 on a numerical pain rating scale) and ability to complete the relevant dose.

##### Gait retraining

The aim of the gait retraining component is to normalise, prevent or minimise gait-related impairments and symptoms that are commonly associated with hip osteoarthritis [28, 53]. This includes pain with walking, reductions in stride length, decreased peak hip extension range of motion, decreased cadence, decreased gait speed, gait asymmetry and increased pelvic obliquity at push-off [27, 28, 53]. Techniques will be prescribed by the trial physiotherapist to normalise stride length and asymmetry. These include, but are not limited to, auditory cueing [54], backwards walking [55, 56] and instructions on techniques that may reduce pain when walking; for example, ‘push more with your feet when you walk’ [57, 58].

##### Motor control (anterior hip stability)

The second phase of the GHOst intervention aims to promote the function of the anterior hip muscles (particularly anterior GMin) using targeted, functional progressions of a split squat and bridge exercise. GMin appears to have a particular vulnerability to atrophy in people with hip pain [26, 32, 37], especially the anterior portion [32, 37–39]. This phase will, therefore, have a focus on anterior GMin muscle recruitment using elastic exercise bands to promote active hip internal rotation [33, 59] in functional, closed-chain positions. Participants will be asked to complete up to five repetitions of 30-s holds for isometric exercises; three sets of 12 to 20 repetitions for dynamic exercises.

##### Pelvic stability with global strengthening

The third phase of the GHOst intervention aims to improve pelvic stability and overall lower-limb and trunk strength and function. These exercises progress from isometric hip hitching [60–63], through to high-intensity resistance-strength training of multiple muscle groups (e.g. double-leg squat and dead-lift) with the aid of Theraband and power-bands (Aussie Strength, Smithfield, NSW Australia) to achieve the required intensity. The high-intensity resistance exercise component of this phase will use the Borg CR10 scale [64–67], asking participants to exercise at a ‘heavy load’ equivalent to Borg ≥ 5 to < 7. This intensity has been validated for use with hip exercises [66]. For the high-intensity resistance exercises, participants will be asked to perform three sets of 6 to 10 repetitions.

### Sham exercise protocol

The sham protocol is similar to that being used in a RCT in people with greater trochanteric pain syndrome [61]. The sham programme differs to the GHOst protocol in that it is not aimed at strengthening the gluteal muscles, but includes generalised lower-limb, low-intensity resistance exercise. Participants are guided through sham gluteal, quadriceps’ and calf exercises, predominantly in a seated position (unloaded).

### Physiotherapy treatment fidelity

To maximise physiotherapy treatment integrity, all trial physiotherapists will have at least 2 years of musculoskeletal clinical physiotherapy experience, and will receive mandatory standardised training (1 day) before being assigned their first participant. Training will cover interventions to be implemented (sham and GHOst), the education to be delivered to participants and trial reporting (e.g. clinical note taking and adverse events). Training will incorporate techniques described by Main et al. [68] for training therapists to implement clinical trial interventions; including trainer-led teaching, role play, group discussions and ongoing clinical mentoring and support throughout the trial.

In addition to training, trial physiotherapists will receive a trial manual that includes an illustrative guide to exercise progressions. Trial physiotherapists will also document the exercises prescribed, adherence, use of pain medication or non-steroidal anti-inflammatory drugs (NSAIDS), and adverse events at each treatment session on standardised recording forms.

### Baseline assessments

Demographic details including age, gender, height and weight will be recorded.

### Outcome measures

The outcome measures are in accordance with those recommended by OARSI for clinical trials on people with hip osteoarthritis [43, 69].

#### Primary outcome measure

##### Self-reported physical function

Self-reported physical function as assessed by the physical function subscale of the Western Ontario and McMaster Universities Osteoarthritis (WOMAC) Index Likert version 3.1 [70].

The physical function subscale of the WOMAC is a 17-item 5-point Likert scale ranging from 0 to 68 with higher scores indicating greater dysfunction. It is a valid and reliable self-administered tool [71] that is recommended for clinical trials in hip osteoarthritis [43], with a minimal clinically important difference of 6 units [72].

#### Secondary outcome measures

##### Physical activity accelerometry

A tri-axial accelerometer (Link; Actigraph Corp., Pensacola, FL, USA) will be used as the objective, reliable measure of physical activity [73]. Participants will attach the accelerometer to a waistband anterior to the right hip for collection of movement data, at an acquisition frequency of 100 Hz. Participants will remove the accelerometers when sleeping (to avoid sleep disturbance) and bathing (due to the lack of water-proofing) [74]. The accelerometer will be worn for a 7-day period in week 0 (prior to commencing the intervention) and week 12 (last week of the intervention).

Output data will be downloaded and analysed in 1-min epoch intervals using the manufacturer’s software (Actilife ver.6; Actigraph Corp., Pensacola, FL, USA). The total time that the participant undertook sedentary, light, moderate and vigorous physical activity will be determined for each valid wear day using previously recognised algorithms [75]. The accumulated total of minutes of moderate and vigorous activity for the week will be summed to determine weekly total moderate to vigorous physical activity (MVPA, min/week) [76].

##### Physical function tests

Recommended OARSI physical function tests for clinical trials on people with hip osteoarthritis [69] will be used, including the 40-m fast-paced walk test, stair-climb test and the chair-stand test. All three tests have acceptable reliability with small measurement error in people with hip osteoarthritis [77], facilitating the ability to measure changes over time.

##### Self-reported physical activity

The International Physical Activity Questionnaire – short form is a self-administered questionnaire that aims to assess health-related physical activity [78]. Participants are asked to recall their physical activity over the last 7 days with regard to vigorous physical activity, moderate physical activity, walking and sitting.

##### Isometric hip-muscle strength

Isometric hip strength will be assessed using handheld dynamometer [79, 80]. Measurement of hip strength using hand-held dynamometry is reliable (ICC_2,1_ = 0.76–0.95) [79, 81]. Hip abduction, adduction, flexion, extension, internal rotation and external rotation will be measured bilaterally (Table 1). The peak force (N) produced from two trials separated by 15 s will be recorded for each action. Participants will be instructed to slowly build up their isometric strength against resistance until they reach their maximum, then maintain this level for 3 s, and slowly relax. Consistent encouragement will be provided via standardised audio recordings. The order of the action tested (e.g. flexion) will be randomly assigned between participants using marked cards that are selected out of a hat, and will remain consistent from pre-test to post-test. Torque (Nm) will be determined (*T* = Force (N) x moment arm (m)) and normalised to body mass (Kg) with the final unit of measurement being Nm/Kg.

**Table 1:** Hip-muscle strength testing protocol

Action	Participant position	Dynamometer placement	Moment arm
Abduction	Supine. Participant holds onto the side of the plinth to stabilise	5 cm proximal to the lateral malleolus	Greater trochanter to 5 cm proximal to lateral malleolus
Adduction	Supine. Contralateral hip and knee flexed, with foot placed flat on the plinth. Participant holds onto the side of the plinth to stabilise	5 cm proximal to the medial malleolus	Greater trochanter to 5 cm proximal to medial malleolus, measured along the lateral surface of the lower limb.
Internal rotation	Sitting over the edge of the plinth. Arms folded across chest	5 cm proximal to the lateral malleolus	Lateral knee joint line to 5 cm proximal lateral malleolus
External rotation	Sitting over the edge of the plinth. Arms folded across chest	5 cm proximal to the medial malleolus	Medial knee joint line to 5 cm proximal medial malleolus, measured along the lateral surface of the lower limb
Hip flexion	Sitting over the edge of the plinth. Arms folded across chest	5 cm proximal to the proximal border of the patella	Greater trochanter to 5 cm proximal to proximal border of patella, measured along the lateral surface of the lower limb
Hip extension	Prone. Testing knee flexed to 90°	Calcaneus	Greater trochanter to Lateral joint line

Note: Resistance applied by the research assistant, with the exception of flexion and extension, where resistance is applied by a belt securing the dynamometer to an external anchor

##### Pain

The average pain over the previous week will be assessed using an 11-point numerical rating scale (NRS), anchored by 0 = ‘no pain’ and 10 = ‘worst pain possible’. The minimal important clinical difference is 1.8 [82].

##### Global hip questionnaires

Hip disability and Osteoarthritis Outcome Score (HOOS) [83, 84]: the HOOS consists of five subscales including (1) pain, (2) symptoms and stiffness, (3) activities of daily living, (4) sport and recreation and (5) quality of life. Participants respond to a 5-point Likert scale for each subscale, and the scores are converted to a 101-point scale, with 100 indicating the best possible score (no difficulty), and 0 indicating extreme symptoms. The HOOS questionnaire is considered reliable (ICC_2,1_ ranging from 0.75 to 0.97) [84].

##### Pain thoughts and depressive symptoms

Pain Catastrophizing Scale (PCS) [85]: the 13-item PCS aims to assess the participants’ thoughts and feelings associated with their pain. Using a 5-point Likert scale, participants are asked to rate the degree to which they have thoughts or feelings related to each item (e.g. ‘I worry all the time about whether the pain will end’). The scale is scored from 0 to 52 with higher scores indicating greater pain catastrophizing, and is associated with higher-intensity physical and emotional stress in response to pain [85]. The PCS is considered a reliable and valid measure of pain catastrophizing [85].

Brief Fear of Movement Scale for osteoarthritis (BFOM) [86]: adapted from the Tampa Scale of Kinesiophobia [87], the BFOM aims to assess the degree to which participants with osteoarthritis feel that physical movement will cause pain, injury or re-injury [86]. The six-item scale is scored from 0 to 24, with a higher score indicating lower fear of movement (better score).

Patient Health Questionnaaire-9 (PHQ-9) [88]: the PHQ-9 is a nine-item valid and reliable scale that aims to measure depression severity. Scored from 0 to 27, participants can be classified as having mild (≥ 5), moderate (≥ 10), moderately severe (≥ 15) and severe (≥ 20) depressive symptoms.

##### Quality of life

Assessment of Quality of Life Questionnaire (AQoL-8D) [89, 90]: the AQoL-8D contains 35 items that assess dimensions of quality of life related to independent living, happiness, mental health, coping, relationships, self-worth, pain and senses. It is a reliable and valid tool [90] that can produce a weighted utility score (0–1), or an unweighted, health-related quality of life score (0–100) with higher scores representing better quality of life.

##### Global change

Global Rating of Change (GROC) [91]: the GROC consists of an 11-point Likert scale that asks participants to rate their perceived overall change in condition of their hip from the beginning of the study. A version of the GROC used in a previous study on hip pain [92] has been adapted for use in this study. A score of ‘0’ will equate to ‘no change’. If ‘better/worse’, participants will be asked to rate the degree of change from ‘slightly better/worse’ to ‘very much better/worse’, with scores ranging from + 1 to + 5 for ‘better’ and − 1 to − 5 for ‘worse’. Scores will further be dichotomised to define ‘success’ as those with a score of ‘moderately better’ to ‘very much better’ (i.e. + 3 or more).

##### Gluteal-muscle activity

Muscle function of the gluteal muscles will be assessed with an EMG in a subset of 30 participants. Fine-wire electrodes will be inserted into GMed and GMin using previously validated procedures [93, 94]. Muscle activity will be recorded during six walking trials across a 10-m pathway. Amplitude and timing variables will be determined during the stance phase of the gait cycle as reported previously [33, 95].

##### Gluteal-muscle size and adiposity

Muscle volume and adiposity will be assessed with MRI in a subset of 30 participants (the same participants undergoing EMG testing). Muscles of interest will include GMin, GMed and gluteus maximus, as well as tensor fascia lata. Muscle volume and adiposity will be determined through off-line processing of de-identified images using customised MATLAB software (The MathWorks, Inc., Natick, MA, USA) [96]. Axial slices of each muscle will be traced to establish cross-sectional area, and multiplied by slice thickness to calculate volume [26, 97]. To determine muscle adiposity, a Muscle-Fat Index (MFI) will be calculated from the axial slices as the proportion of adipose tissue to total muscle (fat/fat + muscle) [98]. This technique has been validated against the ‘gold standard’, biopsy [99]. Given the segmental nature of GMin and GMed, a MFI will be calculated for anterior and posterior regions of each muscle [96].

### Trial follow-up

**Fig. 2:**
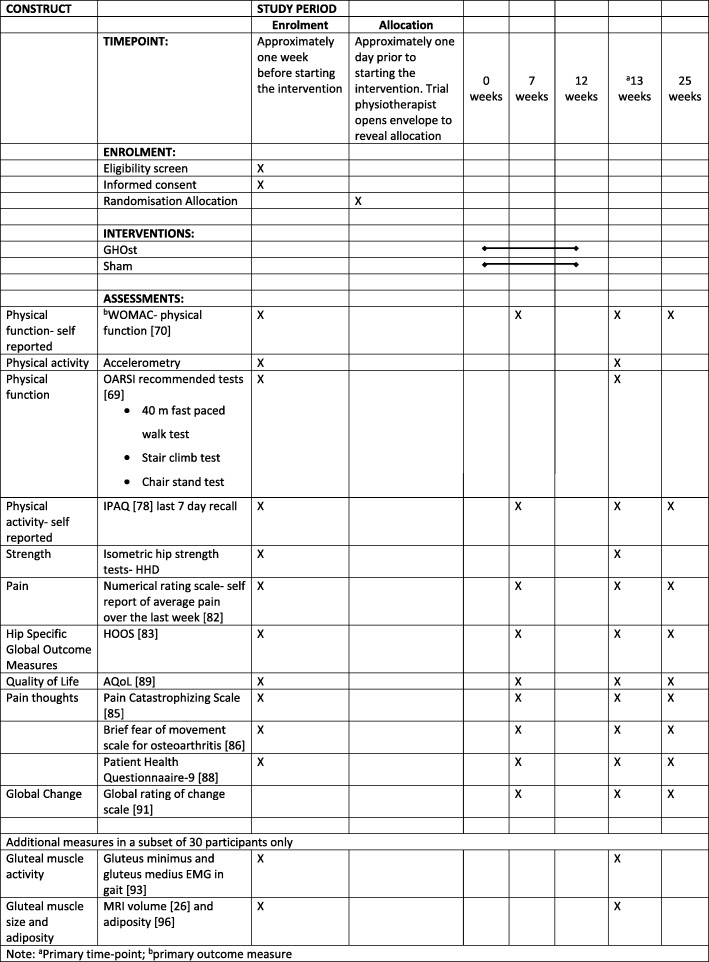
Standard Protocol Items: Recommendations for Interventional Trials (SPIRIT) Checklist: patient schedule of procedures

The primary time-point will be at the completion of the intervention (13 weeks). Additional measures of some outcomes (questionnaires) will be recorded at 7 and 25 weeks. The schedule of enrolment, assessment, intervention and follow-up can be seen in Fig. 2.

### Success of blinding

The success of participant blinding will be assessed 1 week after commencing the intervention by having participants nominate which intervention they feel they have been allocated to (‘generalised exercise programme’ or ‘targeted gluteal programme’ or ‘unsure’. It is not anticipated that large differences in outcome would be present 1 week after commencing the intervention, so the potential benefit (or otherwise) of the intervention will not influence their decision. The validity of assessing the effectiveness of blinding for interventions that may expect large effect sizes is questionable, and is now no longer a mandatory component of the Consolidated Standards of Reporting Trials (CONSORT) Statement [100, 101].

### Adverse events

Adverse events will be monitored and recorded by the physiotherapist and participant. These may include minor aches and pains through to serious adverse events (e.g. myocardial infarction) associated with beginning a new exercise programme.

### Sample size and power analysis

The minimal clinically important difference for the WOMAC is 6 physical function units [72]. Using data from the sham intervention of a previous RCT in people with hip osteoarthritis [21], the standard deviation (SD) of change in the WOMAC over 13 weeks is 9.2 units, assuming a baseline to follow-up correlation of 0.60. A total sample size of 76 participants (38 per group) is required to detect a clinically important difference between sham and intervention groups, with a power of 80% and an alpha of 0.05. A clinically important difference of 6 units between groups, assuming a within-group SD of 9.2 units equates to a moderate to large effect size (0.65). To account for potential drop-outs similar to those reported in previous studies [21] we will recruit 90 participants (45 per group).

### Statistical analysis

Statistical analysis will be using intention-to-treat principles and on a per-protocol basis. Data analysed will focus on detecting the between-group treatment and within-group treatment effects (with effect sizes and 95% confidence intervals) at each of the follow-up time-points. A linear mixed model will be used for the primary analysis of the changes in the WOMAC due to its advantages in modelling the influence of nonlinear, individual differences over time. Adjustments will be made for the respective baseline outcome measures, as well as age, sex and BMI; and adjustments for other baseline variables that have evidence of imbalances between groups (e.g. radiographic severity). The secondary outcomes will be assessed using *t* tests or Mann-Whitney *U* tests, adjusting for baseline differences if required. The Global Rating of Change Scale will be dichotomised to define ‘success’ as those with a score of ‘moderately better’ to ‘very much better’ (i.e. + 3 or more). A generalised mixed model (adjusted for baseline differences and covariates) will be used to assess differences in the proportion of ‘successes’ between groups over each time-point.

## Discussion

This RCT will be the first to investigate the effect of a targeted gluteal rehabilitation programme compared to a sham exercise programme for improving physical function in people with hip osteoarthritis. The targeted GHOst protocol is a structured exercise programme that incorporates gait retraining, motor control and high-intensity resistance exercises. It requires minimal equipment and is implemented by community physiotherapists. This pragmatic design could enable easy integration into any physiotherapy clinical setting. The research design aims to minimise bias associated with selection, measurement and confounding. The results of this study will have significant implications in terms of maximising physical function in people with hip osteoarthritis which may ultimately affect their quality of life.

The GHOst intervention has been specifically developed for people with hip osteoarthritis. Selection of a sample of people with hip osteoarthritis is, therefore, critical to the outcome of this study. We have used an accepted criteria for hip osteoarthritis diagnosis that includes clinical and radiographic confirmation [44]. More recent criteria, for example from the National Institute for Health and Care Excellence (NICE) [13], suggests a clinical diagnosis without the need for radiographic confirmation. Such criteria would enhance generalisability and potentially maximise recruitment rates into the study. However, clinical diagnoses without radiographic confirmation are often paired with higher age thresholds (NICE guidelines; > 45 years) to improve the specificity of the diagnosis. It is increasingly being recognised that osteoarthritis is not just an ‘old-persons’’ disease [102]. A large increase in prevalence of osteoarthritis has been observed in Canadians aged over 35 years (1994–2002 data) [103], and the projected change in healthcare costs associated with osteoarthritis in Australia in those under 65 years is greatest in the 35–44 years age bracket [7]. Radiographic confirmation would, therefore, allow for inclusion of younger people with osteoarthritis, who may have a significant impact on the burden of disease in years to come.

Measurement error and confounding have been limited through the study design. All assessors are blinded to group allocation, and our primary outcomes are valid and reliable. A unique element of the current study is the inclusion of secondary outcomes such as muscle activity (EMG) and muscle size (MRI), which will provide mechanistic evidence of differences (or lack of) between interventions. It is not possible to blind the therapists to group allocation, and this may confound the effects of the intervention if therapists deliver the GHOst intervention with more enthusiasm than the sham. This could potentially lead to inflated effect sizes in favour of the intervention. This will be minimised with appropriate training for therapists on delivery of both interventions with equal enthusiasm.

The results of this study will be disseminated via journal publication and conference presentations. Participants will receive a summary of results via email at the completion of the study. The interventions have been designed to facilitate translation into clinical practice and will aid health and medical practitioners with evidence-based decision-making for the non-pharmacological management of hip osteoarthritis.

## Trial status

This is protocol version 3 (20 October 2017). Recruitment commenced 26 July 2017. Recruitment is expected to be completed by 1 June 2019.

## Additional files

Additional file 1: Standard Protocol Items: Recommendations for Interventional Trials (SPIRIT) 2013 Checklist: recommended items to address in a clinical trial protocol and related documents*. (DOC 135 kb)

Additional file 2: Flow diagram illustrating a clinical diagnosis of gluteal tendinopathy that would warrant exclusion from the proposed study [49]. Abbreviations: *FABER*- flexion, abduction, external rotation test, *OA* osteoarthritis. (DOCX 40 kb)

Additional file 3: Flow diagram illustrating screening for lumbar pathology as the source of hip pain. Abbreviations: *pSLR*  passive straight leg raise, performed to 45° of hip flexion. (DOCX 25 kb)

Additional file 4: Example of the participant information and consent form. Bendigo site. (PDF 184 kb)

## Abbreviations

ANZCTR: Australian and New Zealand Clinical Trials Registry
AQoL-8D: Assessment of Quality of Life Questionnaire-8D
BFOM: Brief Fear of Movement Scale for Osteoarthritis
BMI: Body Mass Index
CONSORT: Consolidated Standards of Reporting Trials
EMG: Electromyography
FABER: Flexion, abduction, external rotation test
GHOst: Gluteal exercises for Hip Osteoarthritis
GMed: Gluteus medius
GMin: Gluteus minimus
GROC: Global Rating of Change
HDEC: Health and Disability Ethics Committee
HOOS: Hip Osteoarthritis Outcome Score
HREC: Human Research Ethics Committee
MFI: Muscle-Fat Index
MRI: Magnetic resonance imaging
MVPA: Moderate to vigorous physical activity
NICE: National Institute for Health and Care Excellence
NRS: Numerical rating scale
NSAIDS: Non-steroidal anti-inflammatory drugs
PCS: Pain Catastrophizing Scale
PHQ-9: Patient Health Questionnaire-9
SD: Standard deviation
SPIRIT: Standard Protocol Items: Recommendations for Interventional Trials
UTN: Universal Trial Number
VAS: Visual analogue scale
WOMAC: Western Ontario and McMaster Universities Osteoarthritis Index
