# Supplementary material for: Targeted gluteal exercise versus sham exercise on self-reported physical function for people with hip osteoarthritis (the GHOst trial – Gluteal exercise for Hip Osteoarthritis): a protocol for a randomised clinical trial
Source: Trials. 2018 Sep 20;19:511. doi: 10.1186/s13063-018-2873-3 (PMC6149073; doi:10.1186/s13063-018-2873-3)
Supplement: Supplementary file 2 — Flow diagram illustrating a clinical diagnosis of gluteal tendinopathy that would warrant exclusion from the proposed study [49]. Abbreviations: FABER- flexion, abduction, external rotation test, OA osteoarthritis. (DOCX 40 kb) [file 13063_2018_2873_MOESM2_ESM.docx]

Exclude from study

Pain reproduction with palpation of greater trochanter

Continue with remaining OA eligibility criteria

Continue with remaining OA eligibility criteria

Continue with remaining OA eligibility criteria

-‘ve

-‘ve

+‘ve

-‘ve

Pain reproduction with at least one of the following clinical tests

- External de-rotation test
- Single leg stance test
- Resisted hip abduction

-‘ve

+‘ve

Pain reproduction with FABER

+‘ve

+‘ve

Continue with remaining OA eligibility criteria

Lateral hip pain
