# Supplementary material for: Targeted gluteal exercise versus sham exercise on self-reported physical function for people with hip osteoarthritis (the GHOst trial – Gluteal exercise for Hip Osteoarthritis): a protocol for a randomised clinical trial
Source: Trials. 2018 Sep 20;19:511. doi: 10.1186/s13063-018-2873-3 (PMC6149073; doi:10.1186/s13063-018-2873-3)
Supplement: Supplementary file 3 — Flow diagram illustrating screening for lumbar pathology as the source of hip pain. Abbreviations: pSLR passive straight leg raise, performed to 45° of hip flexion. (DOCX 25 kb) [file 13063_2018_2873_MOESM3_ESM.docx]

+‘ve

Exclude from study

Hip pain reproduction with any of:

- Repeated lumbar flexion
- Repeated lumbar extension
- Lumbar quadrant

Hip pain reproduction with pSLR

Exclude from study

-‘ve

+‘ve
