# Supplementary material for: Targeted gluteal exercise versus sham exercise on self-reported physical function for people with hip osteoarthritis (the GHOst trial – Gluteal exercise for Hip Osteoarthritis): a protocol for a randomised clinical trial
Source: Trials. 2018 Sep 20;19:511. doi: 10.1186/s13063-018-2873-3 (PMC6149073; doi:10.1186/s13063-018-2873-3)
Supplement: Supplementary file 4 — Example of the participant information and consent form. Bendigo site. (PDF 184 kb) [file 13063_2018_2873_MOESM4_ESM.pdf]

College of Science, Health & Engineering
PO Box 199 | Bendigo Vic 3552
T: 03 5444 7282

Participant Information and Consent Form

La Trobe University and Bendigo Health

Project Title: Can a targeted exercise program improve hip function and increase activity levels in people with hip osteoarthritis; a randomized controlled trial

Investigators: Dr Rodney Green, La Trobe University Bendigo, rod.green@latrobe.edu.au, phone 03 5444 7282

Dr Tania Pizzari, La Trobe University Melbourne, t.pizzari@latrobe.edu.au, phone 03 9479 5872

Dr Adam Semciw, University of Queensland, a.semciw@uq.edu.au, phone 07 3365 4592

Dr Michael Kingsley, La Trobe University Bendigo, m.kingsley@latrobe.edu.au, phone 03 5444 7589

Dr Stephanie Woodley, University of Otago, Dunedin, NZ, stephanie.woodley@anatomy.otago.ac.nz, phone 03 4797 353

Ms Anita Zacharias, La Trobe University Bendigo, a.zacharias@latrobe.edu.au, phone 03 5444 7542

Location: Bendigo site

1. Introduction

You are invited to take part in this research project. This is because you have been diagnosed as having mild to moderate osteoarthritis of the hip and are not excluded because you;

- have other lower limb or lower back musculoskeletal pathology that may refer pain to your hip
- have other conditions or factors that could limit participation in the exercise program (e.g., cardiac conditions)
- are unable to physically complete the testing procedures (e.g., use of walking aid)
- are unable to participate in electromyography (EMG) or Magnetic Resonance Imaging (MRI) testing procedures (e.g., cardiac pacemaker, Bendigo only)

The research project aims to compare two different exercise programs to see which is more effective in improving hip function and activity levels in people with hip osteoarthritis (OA).

This Participant Information and Consent Form tells you about the research project. It explains the tests and treatments involved. Knowing what is involved will help you decide if you want to take part in the research.

Please read this information carefully. Ask questions about anything that you don't understand or want to know more about. Before deciding whether or not to take part, you might want to talk about it with a relative, friend or your GP.

Participation in this research is voluntary. If you don't wish to take part, you don't have to. You will receive the best possible care whether or not you take part.

If you decide you want to take part in the research project, you will be asked to sign the consent section. By signing it, you are telling us that you:

- understand what you have read;
- consent to take part in the research project;
- consent to have the tests and treatments that are described;
- consent to the use of your personal and health information as described.

You will be given a copy of this Participant Information and Consent Form to keep.

2. What is the purpose of this research project?

OA is a common condition, particularly in people over 55 years. Conservative management, including exercise programs are often used as first line treatment for hip OA but many patients eventually have hip replacement surgery as a last resort. Successful rehabilitation programs can improve quality of life through improved hip function and increased activity levels and may delay or negate the need for hip replacement surgery. However, recent studies have confirmed that current rehabilitation programs are limited in terms of improving hip function and reducing pain.

This project will compare two exercise programs to manage hip OA. To date there is no research of one type of exercise program being of benefit over another.

This study is being conducted jointly by La Trobe University, University of Queensland and University of Otago and will recruit 90 participants over 4 sites (Bendigo, Melbourne, Brisbane and Dunedin).

This research is funded by Arthritis Australia and La Trobe University.

3. What does participation in this research project involve?

You will be participating in a randomised controlled research project. Sometimes we do not know which treatment is best for treating a condition. To find out we need to compare different treatments. We put people into groups and give each group a different treatment. The results are compared to see if one is better. If you agree to participate, you will have 50% chance of receiving either treatment, but we are not able to predict which treatment you will receive. Both treatment programs in this study will involve participation in a 12-week exercise program involving weekly visits at a local physiotherapy clinic and testing at 4 time intervals conducted at the University associated with your location, before, during and after the 12-week rehabilitation program. You will be participating in a blind study. In a blind study you do not know which of the treatments you are receiving. Your treating physiotherapist will know which treatment you are receiving.

Before commencing the trial:

Stage 1: phone screening for exclusion criteria already completed.

Stage 2: You will attend an appointment (approximately 30 minutes) with a physiotherapist and an array of clinical tests of your hip will be undertaken to confirm your eligibility for the study. If you meet the criteria for inclusion in the study, further testing will be conducted as described below.

Stage 3: You will be randomly allocated to one of the two different exercise programs. You will not have a choice, nor be informed as to which program you are in.

Rehabilitation programs:

These programs will both involve weekly attendance at a local physiotherapy clinic to be instructed in how to perform the exercises associated with your program. The exercises will be based on an initial private assessment with the physiotherapist and in subsequent sessions may include small group consultations (2-3 participants). You will also be expected to complete exercises at home in between visits to the physiotherapist.

During the period of the trial you will be required not to seek any other active intervention for your hip OA but should continue with your usual medication. You will have the ability to discuss your symptoms during your weekly visits to the physiotherapist and if there is any flare-up of your condition you may be referred back to your usual GP for additional medication if required.

There are no additional costs associated with participating in this research project, nor will you be paid. All physiotherapy care and testing sessions required as part of the research project will be provided to you free of charge.

Testing sessions (weeks 0, 7, 13 & 25):

A series of tests will be conducted before you commence the trial (week 0), during the 12-week exercise program (week 7) and after the exercise program (weeks 13 and 25).

The testing session will be conducted by one of the project investigators at your local University site and take approximately 30 minutes. The testing will involve completion of questionnaires relating to hip function and quality of life, a series of functional tests normally associated with an evaluation for hip osteoarthritis (30-second chair stand, stair climb and 40-metre fast-paced walk tests) and hip muscle strength testing. You will also be given an accelerometer to measure your physical activity over a 7-day period prior to your first/next exercise session appointment with the physiotherapist. You will return the accelerometer to the physiotherapist for data download.

Additional testing at Bendigo site only (weeks 0 and 13)

Testing for hip muscle function is conducted at the University laboratory using electromyography (EMG) and Magnetic Resonance Imaging (MRI) scanning will be conducted at High St Xray to assess hip muscle size. These tests will only be conducted in weeks 0 and 13.

The EMG testing will immediately follow the strength testing session and you will be asked to wear pants with a loose elasticized waist such as bike pants or shorts (a pair will be provided if needed) and comfortable walking/running shoes. Either Dr. Rod Green or Ms. Anita Zacharias will mark and prepare five sites around their hip where intramuscular electrodes will be inserted. The electrodes consist of extremely thin pieces of wire that enable information about muscle activity to be transferred from within the muscles, to a computer in order to be analyzed. The electrodes will be inserted using a needle under the guidance of ultrasound by Dr. Green or Ms. Zacharias. You might experience some discomfort at this point, but this should only be temporary. You will then perform a series of tasks including walking trials and other activities. This session will take up to 2 hours.

At the conclusion of the EMG testing an appointment will be arranged to attend High St Xray for the MRI scan. The technician on site will screen you for contraindications to MRI scanning including metal implants such as a pacemaker. The MRI scan is a painless procedure that involves lying down on your back between in the MRI machine from the feet to chest for approximately 30 minutes. This entire session may take up to 2 hours.

If any abnormal findings of clinical concern (other than normal age-related changes) are identified on your MRI scan, you will be referred to your general practitioner for advice and he/she will receive a copy of the report.

4. Do I have to take part in this research project?

You have volunteered to participate in this research project either by contacting us directly or through your treating practitioner. If you do not wish to take part, you do not have to. If you decide to take part and later change your mind, you have the right to withdraw from active participation in this project at any time and, further, to demand that data arising from your participation are not used in the research project provided that this right is exercised within four weeks of the completion of your participation in the project. You may indicate your desire to withdraw from the project at any time before, during or after testing by contacting the researchers and completing a 'withdrawal of consent' form.

Your decision whether to take part or not, or to take part and then withdraw, will not affect your relationship with the researchers or your usual treating practitioners.

5. What are the possible benefits of participating in this study?

You will receive the following benefits:

- Free clinical assessment of your hip by a physiotherapist
- Free 12-week physiotherapist-supervised exercise program
- Free MRI examination of your hip

We cannot guarantee or promise that you will receive the following benefits from this research; however, possible benefits may include:

- Reduction in hip symptoms (pain and dysfunction)
- Improved function in your hip

6. What are the possible risks of participating in this study?

- *Clinical Hip Tests:* This may cause reproduction of your hip symptoms (e.g. pain, stretch, tightness). This is to ensure correct diagnosis of your hip OA before commencing in the study.
- *Exercise Program:* There are unlikely to be any risk involved in undertaking the exercise program. You may experience some muscle fatigue or tiredness in the muscles being exercised. This is normal muscular response to exercise and should settle within 1- 2 days. The risk of discomfort associated with your exercise program will be closely monitored by your physiotherapist and should you have any questions, you are able to contact your physiotherapist or research investigators at any stage of the study.
- *MRI examination:* There are contraindications for MRI scanning (e.g., cardiac pacemaker) However all participants will be screened prior to commencing the study and again by a technician prior to the scanning procedure.
- *Intramuscular EMG procedures* are considered safe with minimal risk. This test is required to understand when and how the gluteal muscles are functioning. Possible risks include;
    1. Infection – The insertion site may become infected. The risk is minimized through practice of a sterile technique by formally trained investigators
    2. Small wire tip (2 mm in length) may break off the electrode during withdrawal – this is extremely rare and has never occurred in La Trobe University EMG laboratories. Because the very small, inert metal splinter is not harmful and will not cause any adverse health effects, no attempt will be made to remove it.
    3. Feeling unwell during or immediately after electrode insertion –this is usually brief and dealt with by having you seated or lying and in very rare events may require testing to be cancelled.
    4. Increased pain during exercise and bleeding from the insertion site

7. How will I be informed of the final results of this research project?

After completion of study we will be provide you with a summary of the results of this study.

8. What will happen to information about me?

Data files (in a re-identifiable form) will be saved on a password protected research server at each site. Paper documents containing questionnaires completed by the participants will be stored in a locked filing cabinet by the lead investigator at each site (Dr Rod Green at Bendigo). Paper documents will be confidentially shredded after 15 years since of recording data. The computer files containing re-identifiable data (your name will be removed) will be retained for comparison with any future similar studies. Appropriate ethical approval would be sought before the re-use of this data in any future projects.

In any publication and/or presentation or future related research projects, information will be provided in such a way that you cannot be identified. Only grouped (targeted exercise or normal rehabilitation) data will be presented.

9. Can I access research information kept about me?

In accordance with relevant Australian and/or Victorian privacy and other relevant laws, you have the right to access the information collected and stored by the researchers about you. Please contact one of the researchers named at the end of this document if you would like to access your information.

In addition, in accordance with regulatory guidelines, the information collected in this research project will be kept for at least 15 years.

10. Is this research project approved?

The ethical aspects of this research project have been approved by the Human Research Ethics Committees of La Trobe University and Bendigo Health

This project will be carried out according to the National Statement on Ethical Conduct in Human Research (2007) produced by the National Health and Medical Research Council of Australia and Health research Council of New Zealand. This statement has been developed to protect the interests of people who agree to participate in human research studies.

11. Consent

I have read this document and I understand the purposes, procedures and risks of this research project as described within it.

I have had an opportunity to ask questions and I am satisfied with the answers I have received.

I freely agree to participate in this research project, as described.

I understand that I will be given a signed copy of this document to keep.

Participant's name (printed) …………………………………………………

Signature Date

Declaration by researcher*: I have given a verbal explanation of the research project, its procedures and risks and I believe that the participant has understood that explanation.

Researcher's name (printed) …………………………………………………

Signature Date

12. Who can I contact if I have any questions?

The person you may need to contact will depend on the nature of your query. Therefore, please note the following:

For further information or appointments:

If you want any further information concerning this project or if you have any problems which may be related to your involvement in the project (for example, feelings of distress), you can contact the principal researchers;

Dr Rodney Green, La Trobe University Bendigo, rod.green@latrobe.edu.au, phone 03 5444 7282

Ms Anita Zacharias, La Trobe University Bendigo, a.zacharias@latrobe.edu.au, phone 03 5444 7542

For complaints:

If you have any complaints about any aspect of the project, the way it is being conducted or any questions about being a research participant in general, then you may contact:

Secretary, Human Ethics Committee, La Trobe University, humanethics@latrobe.edu.au, phone 9479 1443

OR

Sally McCarthy, HREC Secretary, Bendigo Health, Sally McCarthy SAMcCarthy@bendigohealth.org.au, phone 5454 6412
